# Beyond dietary therapy: addressing weight stigma awareness in medical students

**DOI:** 10.1186/s12967-025-06981-7

**Published:** 2025-08-22

**Authors:** Giuseppe Annunziata, Giordano Bruno Zonzini, Evelyn Frias-Toral, Raynier Zambrano-Villacres, Alexander Bertuccioli, Davide Sisti, Ludovica Verde, Daniele Di Pauli, Annamaria Colao, Giovanna Muscogiuri, Daniel Simancas-Racines, Alessandro Gennaro, Luigi Barrea

**Affiliations:** 1 Facoltà di Scienze Umane, della Formazione e dello Sport, Università Telematica Pegaso, Via Porzio, Centro Direzionale, Isola F2, 80143 Naples, Italy; 2 Department of Biomolecular Sciences, University of Urbino Carlo Bo, Via Aurelio Saffi 2, 61029 Urbino, Italy; 3 School of Medicine, Universidad Católica de Santiago de Guayaquil, Av. Pdte. Carlos Julio Arosemena Tola, 090615 Guayaquil, Ecuador; 4 Division of Research, Texas State University, 601 University Dr, San Marcos, TX 78666 USA; 5 Escuela de Nutrición y Dietética, Universidad Espíritu Santo, 0901952 Samborondón, Ecuador; 6 Department of Public Health, University of Naples Federico II, Via Sergio Pansini 5, Naples, Italy; 7 Independent Researcher, 38068 Rovereto, Italy; 8 Unità Di Endocrinologia, Dipartimento Di Medicina Clinica E Chirurgia, Università Degli Studi Di Napoli Federico II, Naples, Italy; 9 Dipartimento Di Endocrinologia, Diabetologia, Andrologia e Nutrizione, Centro Italiano Per La Cura E Il Benessere del Paziente Con Obesità (C.I.B.O), AOU Federico II, Via Sergio Pansini 5, 80131 Naples, Italy; 10 Cattedra Unesco “Educazione Alla Salute E Allo Sviluppo Sostenibile”, University Federico II, Naples, Italy; 11 Facultad de Ciencias de la Salud Eugenio Espejo, Centro de Investigación en Salud Pública y Epidemiología Clínica (CISPEC), Universidad UTE, 170527 Quito, Ecuador; 12 Dipartimento Di Psicologia E Scienze Della Salute, Centro Direzionale, Università Telematica Pegaso, Via Porzio, Isola F2, 80143 Naples, Italy

**Keywords:** Weight stigma, Obesity, Healthcare students, University programs, Inclusive attitudes

## Abstract

**Background:**

The growing prevalence of obesity worldwide has drawn increased attention to the issue of weight stigma. Discriminatory attitudes related to body weight are evident across various settings, including those that should ideally offer support to individuals with obesity, such as schools and sports facilities. This research aimed to examine weight-related stigma among university students enrolled in healthcare-related academic programs.

**Methods:**

To measure weight stigma, researchers administered the Italian version of the Attitude Toward Obese Persons (I-ATOP) questionnaire to 201 students from the University of Urbino (Italy) and the University of Malaga (Spain). The analysis explored variations in stigma levels based on gender, BMI classification, nationality, Italian regional location, level of academic education, medical history, and lifestyle factors.

**Results:**

The participant pool was predominantly female (58.2%) and Spanish (66.7%), with an average age of 22.86 ± 3.08 years and a mean BMI of 22.80 ± 3.25 kg/m^2^, placing most respondents in the normal weight range. Nearly half (47.8%) demonstrated a low degree of stigmatising attitudes. Within the sample, significantly lower stigma levels were reported among female and Spanish students (*p* = 0.001 and *p* = 0.011, respectively), as well as among those without a history of eating disorders (*p* = 0.017) and those who engaged in physical activity (*p* = 0.029). Additionally, stigma showed a notable decline in relation to higher educational attainment (*p* = 0.010).

**Conclusion:**

This pilot study reveals the presence of weight stigma even within healthcare education settings, where future health professionals are being trained. These findings underscore the urgent need for comprehensive educational strategies aimed at fostering inclusive and nonjudgmental attitudes toward individuals with obesity. Importantly, the assessment and management of weight stigma should extend beyond nutritional therapy alone, recognizing its psychological, social, and structural dimensions. Integrating this broader perspective into healthcare curricula is thus essential to improve the quality of care for individuals living with one of the most prevalent and complex chronic conditions.

## Background

Weight stigma encompasses a range of negative stereotypes and prejudicial attitudes, targeted at individuals based on their body weight and size. It can manifest in various forms, including persistent teasing, verbal harassment, overt hostility, and instances of discrimination and bullying [1]. Individuals affected by weight stigma are frequently stereotyped as lazy, lacking self-discipline, or incapable of managing their health, despite these assumptions being largely unfounded and rooted in cultural biases rather than scientific evidence [2]. In this context, a concerning issue is that, as the global prevalence of obesity continues to rise [3], weight stigma is simultaneously becoming increasingly pervasive [4], posing a significant threat to the health of individuals with obesity and potentially undermining the effectiveness of therapeutic interventions.

Weight stigma is largely rooted in a reductive understanding of obesity, often perceived as a condition resulting solely from a lack of personal willpower. This narrow perspective perpetuates stigmatising attitudes and contributes to inconsistencies in clinical management. It also reinforces an overestimation of the efficacy of lifestyle-based interventions, while delaying the timely adoption of more effective treatment modalities, such as pharmacotherapy and bariatric surgery [5]. Concurrently, weight stigma has detrimental effects on both mental and physical health, increasing vulnerability to conditions such as depression, anxiety, low self-esteem, negative body image, and maladaptive eating behaviours (e.g., binge eating, resistance to dietary guidance). It also contributes to a diminished quality of life, further weight gain, and reduced engagement in physical activity [6].

Given the widespread nature of weight stigma, it is essential to investigate potential predisposing factors and assess its prevalence within specific population subgroups, in order to inform the development of targeted awareness and prevention strategies. Current evidence suggests that weight stigma may not be strongly associated with sex differences, as several studies have reported comparable levels of stigma among males and females [7, 8]. Conversely, age appears to be a more influential factor, with older individuals exhibiting more negative attitudes toward obesity [8].

Educational attainment has also been considered in shaping stigmatising attitudes; however, its exact role remains ambiguous [9]. Indeed, the literature presents conflicting findings, with some studies identifying a protective effect of higher education [10] while others suggest the opposite association [11]. Although these inconsistencies may be partly attributed to methodological differences in stigma assessment, they highlight the necessity for further research to clarify the relationship between education and weight stigma. Such efforts are critical to delineate the trajectory of this phenomenon and to inform the design of effective educational interventions aimed at mitigating stigma.

It is particularly concerning that weight stigma might be prevalent even in environments that should serve as psychologically supportive spaces for individuals with obesity. Notably, research indicates that individuals who perceive or experience weight-related stigma often tend to avoid engaging in physical activity and reduce participation in sports [12, 13], as a self-protective measure to minimize exposure to stigmatisation within such settings [14]. Consequently, affected individuals may prefer to exercise in isolation or in less populated environments, further reflecting how stigma negatively influences physical activity behaviours [14]. These findings underscore a troubling paradox: the realm of sport, typically associated with inclusion and health promotion, may instead serve as a setting where individuals with obesity face exclusion and discrimination [15].

This pattern of stigmatisation is not confined to sports or physical activity settings. Remarkably, similar dynamics are frequently observed in other domains, where support and care are expected and, most notably, in healthcare environments. Despite expectations to the contrary, weight stigma is also widely documented within healthcare settings [16, 17]. A recent Italian study exploring the prevalence of weight stigma revealed that nearly all participants with obesity (98%) reported experiencing stigmatisation at different stages of life, highlighting the extensive nature of the issue. Notably, schools (86.94%) and healthcare environments (80.93%) were among the most frequently cited settings for stigma, with 80% of respondents reporting stigmatising experiences from healthcare professionals and 56.34% from educators [18]. Additionally, research from the UK and the US indicates that students in dietetics programs may exhibit similar levels of stigmatising attitudes toward individuals with obesity as their peers in non-health-related disciplines [19, 20].

Collectively, these findings highlight the widespread nature of weight stigma within both educational and healthcare settings, underscoring the imperative to implement targeted training programs for future professionals in these sectors, particularly those specializing in nutrition. Building on this context, the present study aims, for the first time, to evaluate weight-related stigmatising attitudes among university students enrolled in healthcare programs across European countries. The investigation specifically focuses on examining the influence of factors such as sex, educational attainment, geographical region, and engagement in physical activity. Importantly, these efforts reaffirm that addressing weight stigma must extend beyond the scope of nutritional therapy alone; a comprehensive approach that includes psychological, social, and structural dimensions is essential to effectively reduce stigma and improve health outcomes for individuals living with obesity.

## Material and methods

### Study participants

This study involved 201 male and female students enrolled in medical, healthcare, and dietetics programs at the University of Urbino (Italy) and the University of Malaga (Spain). Participants were recruited through classroom announcements by faculty members. Prior to enrolment, all participants were provided with an information sheet outlining the study’s aims and procedures. Each participant provided signed informed consent.

Participants self-reported their sociodemographic and personal data, including gender, age, weight, height, nationality, geographical location, educational level, family history of obesity, history of eating disorders, physical activity habits, and dietary patterns, using a standardised questionnaire. The stigma assessment questionnaire was administered to each participant by trained staff.

Body mass index (BMI) was calculated from self-reported weight (kg) and height (m) using the formula weight divided by height squared (kg/m^2^). Participants were classified according to the World Health Organization BMI categories [21]: underweight (≤ 18.49 kg/m^2^), normal weight (18.5–24.9 kg/m^2^), overweight (25.0–29.9 kg/m^2^), and obesity (≥ 30.0 kg/m^2^).

For the regional analysis, Italian students were grouped according to their region of residence into the following categories: North Italy, Central Italy, South Italy, and Islands (Sicily and Sardinia). Educational attainment was categorised as: high school diploma (students enrolled in a Bachelor’s degree program), Bachelor’s degree (students enrolled in Master’s degree programs), and Master’s degree (students attending postgraduate courses).

Incomplete questionnaires (i.e., those with missing responses on any of the key variables used in the analysis) were excluded from the final sample. The study protocol was approved by the Local Ethics Committee of Università Telematica Pegaso, under resolution no. PROT./E 005082, dated 19 July, 2024.

### Weight stigma assessment

Stigmatising attitudes among students were assessed using the Italian version of the Attitudes Toward Obese Persons (I-ATOP) validated questionnaire [22], a widely recognised psychometric tool for measuring individuals’ attitudes toward people with obesity. The I-ATOP consists of 16 items, each rated on a 6-point Likert scale. Higher total scores correspond to more positive and less stigmatising attitudes, whereas lower scores indicate stronger weight-related bias and stigma [23].

Scoring involves three steps: (i) reverse-coding negatively worded items, (ii) summation of all item scores, and (iii) addition of a constant value of 60 to the total score.

The final score ranges from 0 to 120, with lower values denoting more negative attitudes toward individuals with obesity.

As outlined by Zagaria et al. [22], the questionnaire includes the following individual items (some of which have been linguistically optimised to avoid stigma, but without altering the original meaning): (1) People with obesity are just as happy as people without obesity. (2) Most people with obesity feel that they are not as good as others. (3) Most people with obesity are more self-conscious than others. (4) Workers with obesity cannot be as successful as other workers. (5) Most people without obesity would not want to marry someone with obesity. (6) People with severe obesity are usually untidy. (7) People with obesity are just as self-confident as others. (8) Most people feel uncomfortable when associating with individuals with obesity. (9) Most people with obesity have different personalities from those without obesity. (10) Most people with obesity feel resentment toward people without obesity. (11) People with obesity are more emotional than people without obesity. (12) People with obesity should not expect to live normal lives. (13) People with obesity are just as healthy as those without obesity. (14) People with obesity are just as sexually attractive as those without obesity. (15) People with obesity tend to have family problems. (16) One of the worst things that could happen to a person is to develop obesity.

### Statistical analysis

Data are presented as means ± standard deviation (SD) or as frequencies, including both absolute counts and percentages. Differences between two groups (e.g., sex: male vs. female; nationality: Italy vs. Spain; history of obesity: yes vs. no; obesity familiarity: yes vs. no; history of eating disorders: yes vs. no; physical activity: yes vs. no; dietary history: yes vs. no) were assessed using unpaired t-tests for independent samples. Comparisons among multiple groups (BMI categories, geographical regions, and education levels) were conducted using one-way analysis of variance (ANOVA) followed by Bonferroni post hoc tests to adjust for multiple comparisons.

Associations between weight stigma, as measured by I-ATOP scores, and study variables were evaluated using Proportional Odds Ratio (OR) models, along with 95% confidence intervals (CIs) and coefficient of determination (R^2^). Statistical analyses were performed using IBM SPSS Statistics (PASW Version 21.0, SPSS Inc., Chicago, IL, USA) and MedCalc^®^ software (version 12.3.0, MedCalc Software bvba, Mariakerke, Belgium). Data visualisation was generated using version 9.1.1 of GraphPad Prism.

## Results

### Descriptive analysis of the study participants

A total of 201 university students (84 males and 117 females) participated in the study. As summarised in Table 1, the mean age was 22.87 ± 3.08 years. The average BMI indicated a normal weight status (22.80 ± 3.25 kg/m^2^). The mean I-ATOP stigma score among participants was 66.56 ± 13.65.

**Table 1:** Participant demographics, anthropometrics, and stigma scores

Parameters	N	Mean	SD
Age (years)	201	22.86	3.08
Weight (kg)	201	66.33	13.22
Height (m)	201	1.70	0.86
BMI (kg/m^2^)	201	22.80	3.25
Stigma score	201	66.56	13.65

Data are expressed as mean ± standard deviation. BMI, body mass index; SD, standard deviation

The distribution of categorical variables is detailed in Table 2. Most participants were Spanish nationals (66.7%), while most Italian students were from Central Italy (58.2%). Regarding educational attainment, 32 students (15.9%) had completed high school, 84 (41.8%) were pursuing a Bachelor’s degree, and 85 (42.3%) were enrolled in Master’s degree programs.

**Table 2:** Frequency distribution of sex, BMI, nationality, geographical area, educational level, clinical history, and lifestyle habits among university students

Parameters	N	%
*Sex*		
Males	84	41.8
Females	117	58.2
*BMI categories*		
Underweight	6	3.0
Normal weight	156	77.6
Overweight	34	16.9
Obesity	5	2.5
*Nationality*		
Italy	67	33.3
Spain	134	66.7
*Geographical area (Italy)*		
North	19	28.4
Center	39	58.2
South	5	7.5
Islands	4	6.0
*Educational qualification obtained*		
High school degree	32	15.9
Bachelor’s degree	84	41.8
Master’s degree	85	42.3
*Obesity history (Did you suffer from obesity?)*		
No	183	91.0
Yes	18	9.0
*Obesity familiarity*		
No	128	63.7
Yes	73	36.3
*Eating disorders history (Did you suffer from eating disorders?)*		
No	170	84.6
Yes	31	15.4
*Physical activity practice*		
No	51	25.4
Yes	150	74.6
*Dietary history (Did you follow a nutritional plan?)*		
No	104	51.7
Yes	97	48.3

Data are expressed as number (N) and percentage (%). BMI: body mass index

Within the total sample, the majority reported no personal history of obesity (91%) or family history of obesity (63.7%), did not have an eating disorder diagnosis (84.6%), reported engaging in regular physical activity (74.6%), and did not follow a specific dietary regimen (51.7%).

### Frequency distribution and analysis of stigmatising attitudes

The frequency distribution of responses to the 16 items of the I-ATOP questionnaire is presented in Table 3. A significant sex-based difference was observed: females exhibited higher I-ATOP scores than males (69.29 ± 14.45 vs. 62.77 ± 11.51; *p* = 0.001), indicating that males expressed more stigmatising attitudes than females (Table 4).

**Table 3:** Frequency distribution of responses to the 16 items of the italian version of the attitudes toward obese persons (I-ATOP) questionnaire

Item	Completely disagree	Moderately disagree	Slightly disagree	Slightly agree	Moderately agree	Completely agree
	N, %	N, %	N, %	N, %	N, %	N, %
1	18, 9.0	43, 21.4	50, 24.9	33, 16.4	42, 20.9	15, 7.5
2	6, 3.0	20, 10.0	28, 13.9	63, 31.3	67, 33.3	17, 8.5
3	7, 3.5	6, 3.0	17, 8.5	42, 20.9	81, 40.3	48, 23.9
4	86, 42.8	42, 20.9	29, 14.4	32, 15.9	8, 4.0	4, 2.0
5	36, 17.9	31, 15.4	36, 17.9	58, 28.9	28, 13.9	12, 6.0
6	72, 35.8	39, 19.4	35, 17.4	28, 13.9	16, 8.0	9, 4.5
7	28, 13.9	59, 29.4	56, 27.9	30, 14.9	20, 10.0	8, 4.0
8	87, 43.3	53, 26.4	31, 15.4	17, 8.5	13, 6.5	0, 0
9	68, 33.8	47, 23.4	39, 19.4	27, 13.4	11, 5.5	7, 3.5
10	60, 29.9	58, 28.9	40, 19.9	32, 15.9	8, 4.0	3, 1.5
11	52, 25.9	45, 22.4	44, 21.9	39, 19.4	14, 7.0	6, 3.0
12	99, 49.3	36, 17.9	28, 13.9	14, 7.0	14, 7.0	9, 4.5
13	106, 52.7	52, 25.9	24, 11.9	8, 4.0	6, 3.0	5, 2.5
14	44, 21.9	55, 27.4	40, 19.9	24, 11.9	22, 10.9	16, 8.0
15	52, 25.9	42, 20.9	41, 20.4	34, 16.9	28, 13.9	3, 1.5
16	83, 41.3	46, 22.9	33, 16.4	14, 7.0	17, 8.5	8, 4.0

Data are expressed as number (N) and percentage (%)

**Table 4:** Differences in weight stigma levels according to sex, bmi, nationality, geographical area, educational level, clinical history, and lifestyle habits among university students

Parameters	Mean	SD	*p* value
*Sex*			
Males	62.77	11.51	**0.001**
Females	69.29	14.45	
*BMI categories*			
Underweight	69.83	18.00	0.583
Normal weight	67.08	13.29	
Overweight	64.12	14.55	
Obesity	63.20	15.14	
*Nationality*			
Italy	63.27	12.20	**0.011**
Spain	68.22	14.08	
*Geographical area (Italy)*			
North	67.00	12.97	0.318
Center	61.43	11.88	
South	66.80	10.57	
Islands	59.00	12.35	
*Educational qualification obtained*			
High school degree	60.62	12.34	**0.010**
Bachelor’s degree	66.24	13.93	
Master’s degree	69.13	13.26	
*Obesity history (Did you suffer from obesity?)*			
No	66.59	13.51	0.999
Yes	66.28	15.46	
*Obesity familiarity*			
No	67.87	13.68	0.073
Yes	64.29	13.39	
*Eating disorders history (Did you suffer from eating disorders?)*			
No	67.54	12.67	**0.017**
Yes	61.22	17.41	
*Physical activity practice*			
No	70.18	16.66	**0.029**
Yes	65.34	12.29	
*Dietary history (Did you follow a nutritional plan?)*			
No	67.10	12.49	0.571
Yes	66.00	14.85	

Data are expressed as mean ± standard deviations. *p* Values in bold denoted statistical significance (*p* < 0.05). BMI, body mass index; SD, standard deviation

No statistically significant variation in stigma scores was found across BMI categories (*p* = 0.583), although minor differences were noted between groups (I-ATOP score = 69.83, 67.08, 64.12, 63.20, underweight, normal weight, overweight, obesity, respectively) (Table 4).

Regarding nationality, a significant difference emerged: Italian students scored lower on the I-ATOP than their Spanish counterparts (63.27 ± 12.20 vs. 68.22 ± 14.08; *p* = 0.011), suggesting stronger stigmatising attitudes among Italian students (lower score = more stigma) (Table 4). No significant differences in stigma levels were detected across different Italian geographical regions (*p* = 0.318).

Similarly, no significant differences in I-ATOP scores were found based on personal history of obesity (*p* = 0.999), family history of obesity (*p* = 0.073), or dietary history (*p* = 0.571). However, students with a history of eating disorders (*p* = 0.017) and those practising physical activity (*p* = 0.029) demonstrated higher levels of stigma compared to their respective counterparts (Table 4).

**Fig. 1:**
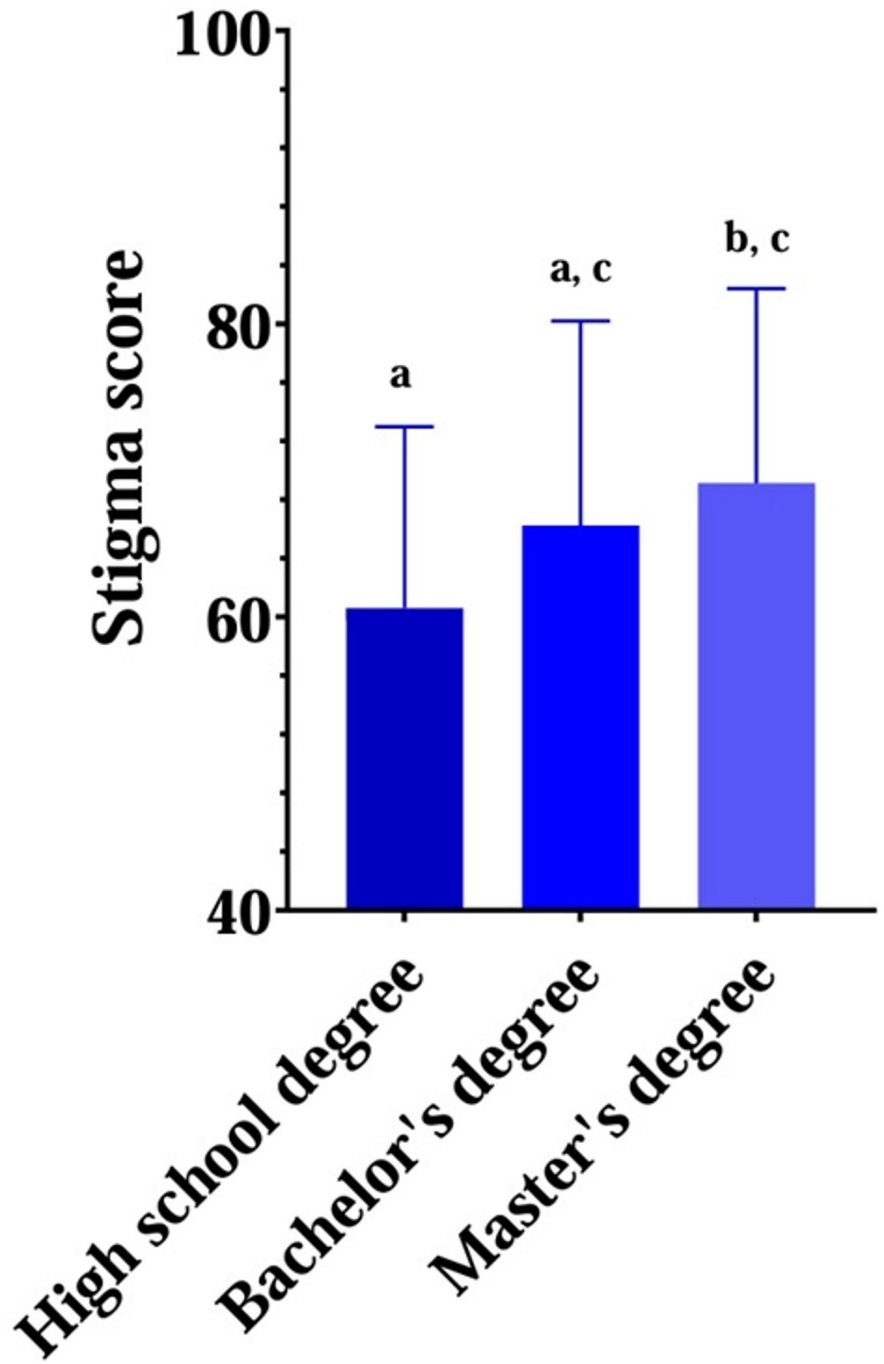
Differences in Stigma Levels According to Educational Attainment. Data are expressed as mean ± standard deviations. The average Italian Version of the Attitudes Toward Obese Persons (I-ATOP) 16-Item Questionnaire were: High school degree = 60.62 ± 12.34, Bachelor’s degree = 66.24 ± 13.93, Master’s degree = 69.13 ± 13.26. Differences between the three groups were analysed by ANOVA test followed by Bonferroni post hoc analysis. Letters refer to the differences between groups (a = High school degree *vs* Bachelor’s degree, *p* = 0.136; b = High school degree *vs* Master’s degree, *p* = 0.008; c = Bachelor’s degree *vs* Master’s degree, *p* = 0.488)

Stigmatising attitudes also varied significantly according to education level (*p* = 0.010). Students with only a high school diploma showed the lowest mean I-ATOP scores (60.62 ± 12.34), whereas those with a Master’s degree scored the highest (69.13 ± 13.26) (Table 4, Fig. 1). Bonferroni post hoc analysis confirmed that students enrolled in Master’s programs exhibited significantly more positive attitudes than Bachelor’s degree students (*p* = 0.008), while no significant differences were found between Bachelor’s degree graduates and either Bachelor’s students (*p* = 0.136) or Master’s graduates (*p* = 0.488). These findings suggest an association between lower educational attainment and increased weight stigma.

A bivariate proportional odds ratio model was conducted to evaluate associations between weight stigma and study variables. Significant associations were found between weight stigma and sex (*p* = 0.001), nationality (*p* = 0.017), education level (*p* = 0.009 for high school degree; *p* = 0.024 for Master’s degree), history of eating disorders (*p* = 0.020), and physical activity practice (*p* = 0.030) (Table 5).

**Table 5:** Bivariate odds ratio model assessing the association between weight stigma and sociodemographic, clinical, and lifestyle factors

Parameters	OR	*p value	95% IC	R^2^
Sex	1.04	**0.001**	1.02–1.06	0.055
Nationality	1.03	**0.017**	1.00–1.05	0.029
*Geographic area (Italy)*				
North	1.03	0.120	0.99–1.09	0.038
Center	0.97	0.150	0.93–1.01	0.032
South	1.02	0.500	0.95–1.11	0.007
Islands	0.97	0.470	0.89–1.05	0.008
*Educational qualification*				
High school degree	0.96	**0.009**	0.94–0.99	0.035
Bachelor’s degree	0.99	0.772	0.98–1.02	0.001
Master’s degree	1.03	**0.024**	1.00–1.05	0.026
*BMI categories*				
Underweight	1.02	0.551	0.96–1.08	0.002
Normal weight	1.01	0.318	0.99–1.04	0.005
Overweight	0.98	0.252	0.96–1.01	0.007
Obesity	0.98	0.576	0.92–1.05	0.002
Obesity history	0.99	0.925	0.96–1.03	0.001
Obesity familiarity	0.98	0.076	0.96–1.00	0.016
Eating disorders history	0.96	**0.020**	0.94–0.99	0.027
Physical activity practice	0.97	**0.030**	0.95–0.99	0.024
Dietary history	0.99	0.569	0.97–1.02	0.002

*A *p-*value in bold type denotes a significant difference (*p* < 0.05)

OR, Odds Ratio; IC, confidence interval; BMI, body mass index

## Discussion

This study provides, for the first time, an overview of weight stigma levels among students enrolled in medical, healthcare, and nutrition university programs across two European countries. The findings reveal differences associated with sex, nationality, educational level, history of eating disorders, and physical activity. Several key observations emerge from this analysis.

Despite the limited sample size, a statistically significant difference in stigma levels was found between students from Spain and Italy, with Italian students exhibiting more stigmatising attitudes than their Spanish counterparts. To date, there is scarce research comparing stigma attitudes among future healthcare professionals from these two countries, and the present pilot study’s small sample size limits the strength of comparative conclusions; thus, these results should be interpreted as descriptive. Similarly, the cross-sectional design does not allow causal links to be deduced.

Existing literature underscores the widespread nature of weight stigma in Italy. For example, a recent study reported that nearly 98% of individuals with obesity have experienced stigma at some point in their lives [18]. Nonetheless, to our knowledge, no prior research has examined regional variations in stigmatising attitudes within Italy. Our results suggest no significant differences across the Italian geographical regions (North, Centre, South, Islands), reinforcing the notion that weight stigma is a pervasive social phenomenon that transcends geographic boundaries.

Few studies have explored whether sex influences the degree of weight stigma. Contrary to the findings of our cohort, which showed that male students exhibited higher levels of stigma compared to females, most prior studies have not reported significant sex-based differences in weight-related stigmatising attitudes. For instance, a survey of 583 participants found no sex-based differences in weight stigma as measured by the Fat-Phobia Scale, suggesting comparable levels of negative bias between males and females [7]. Similarly, studies employing the I-ATOP scale have identified age, rather than sex, as a significant factor affecting weight stigma, with older individuals tending to exhibit more negative attitudes; thereby, suggesting a potential moderating role of educational exposure or life stage [8].

In contrast to some previous findings, our results indicate that students with the lowest education level (high school degree) exhibited higher levels of weight stigma compared to those pursuing or holding a Master’s degree. The influence of education on weight stigma remains ambiguous, as previous studies have reported conflicting evidence. For instance, a multicentre study by Puhl et al. involving 2886 participants found that higher educational attainment correlated with increased levels of weight stigma [10]. In contrast, another investigation employing vignettes and semantic differential scales reported an inverse relationship, where higher education was linked to lower stigma levels [11]. Similarly, a recent systematic review exploring the association between educational attainment and stigmatising attitudes toward individuals with obesity highlighted inconsistent findings, leaving the role of education inconclusive [9].

In this sample, our findings suggest a more nuanced interaction between education and stigma, potentially shaped by multiple influencing factors. The reduced stigma observed among participants with higher education may reflect greater awareness and understanding of obesity, fostering more empathic and less judgemental attitudes. Additionally, individuals with advanced education are also more likely to possess enhanced health literacy and a deeper comprehension of obesity’s complex etiopathogenesis, which may contribute to diminished stigmatising perceptions.

These observations support the hypothesis that weight stigma may, at least in part, arise from inadequate health literacy and limited knowledge about obesity. Although it is not possible to establish a causal link between the level of education and stigma, higher educational attainment often exposes individuals to diverse viewpoints and social issues, promoting social inclusion. Nevertheless, societal and cultural contexts may also influence how education impacts attitudes toward obesity, underscoring the multifactorial nature of this relationship.

Our study underscores a concerning issue regarding the presence of weight stigma among future healthcare professionals, particularly those specialising in nutrition. These results are consistent with a prior survey involving 2449 women with obesity, which identified healthcare professionals as one of the primary sources of stigma, second only to family members [24]. The perception of judgmental attitudes related to body weight in healthcare settings may lead individuals to avoid, delay, or cancel routine screenings and essential treatments. Moreover, such stigma can erode the therapeutic alliance between patient and provider, increasing the risk of condition recurrence.

Supporting this, previous research has shown that weight-based stigmatising attitudes in clinical obesity care are a leading cause for postponement or cancellation of medical appointments [25]. Consequently, stigma within healthcare environments can compromise treatment efficacy and discourage patients from seeking necessary care, thereby worsening obesity-related health outcomes.

To date, no studies have specifically assessed the prevalence of stigmatising attitudes among future nutrition professionals in Italy. Existing evidence is limited to two earlier studies conducted with dietetics students in the UK and USA, which found that these students were neither more nor less susceptible to weight stigma than their peers in other academic disciplines. However, these studies may not fully reflect the evolving sociocultural dynamics currently influencing weight stigma, highlighting the need for updated investigations in this population [19, 20].

Another notable finding from our study was that students who engage in physical activity exhibited significantly higher levels of weight stigma compared to their non-active counterparts. While physical exercise is widely recognised as a cornerstone intervention in obesity management, it may paradoxically serve as a barrier to treatment by acting as a source of weight stigma. Specifically, this suggests that involvement in physical activity may be linked to more negative attitudes toward individuals with overweight or obesity, potentially undermining the positive effects of exercise on health outcomes in this population. To the best of our knowledge, there is currently no solid evidence available regarding the stigmatising attitudes of people engaged in sports toward individuals with obesity. Conclusions on our findings, therefore, remain purely speculative. One possible explanation is that participation in sports and fitness contexts (often idealised as environments of discipline, performance and physical aesthetics) may reinforce social norms that value thinness and/or emphasise weight loss and body control, tending to stigmatise larger body types and inadvertently contributing to distorted attitudes. Individuals immersed in these environments may internalise appearance-based standards and adopt a more critical view of those who do not conform. These aspects, moreover, may further influence how physically active students perceive individuals with obesity, especially in Western societies where physical fitness is equated with moral virtue. Although research exploring the interplay between physical activity and weight stigma remains limited [10], several studies involving adolescents and university students have demonstrated that perceiving or experiencing weight stigma correlates with an increased tendency to avoid physical activity and a reduction in exercise participation across different intensities [12, 13]. This avoidance effect appears to be particularly pronounced among females [26] and may be further intensified by the internalisation of stigmatising attitudes [27]. Recent evidence also suggests that individuals with obesity may adopt coping strategies by either avoiding or selectively managing their engagement in physical activity to minimize exposure to stigmatising experiences within exercise environments [14]. Consequently, our findings corroborate prior research linking sports and physical exercise settings with elevated levels of weight stigma and discrimination [15]. Although physical activity remains essential in obesity treatment, individuals affected by obesity may perceive participation in sports as a potential “threat” due to the risk of encountering stigma, which can lead to avoidance behaviours and thus exacerbate the obesity condition.

### Limitations and strengths

The primary strength of our study lies in its novel assessment of weight stigma among university students enrolled in medical, healthcare, and nutrition programs across European countries. To our knowledge, this is the first investigation to evaluate differences in stigmatising attitudes according to sex, BMI categories, nationality, geographical area, educational level, clinical history, and lifestyle factors within this population. Overall, the study provides valuable insights into the pervasive nature of weight stigma, underscoring its presence even among future healthcare and nutrition professionals in Europe and Italy. Moreover, it highlights the occurrence of stigma within sports environments, which may contribute to the avoidance of physical exercise among individuals with obesity. Another notable strength is the identification of the positive influence of health education and cultural knowledge in mitigating weight stigma. However, several limitations must be acknowledged. The relatively small sample size restricts the generalizability of our findings. The unbalanced distribution of participants between countries (particularly the overrepresentation of Spanish students compared to Italian students) may have biased the national comparisons and limited the reliability of between-country analyses. Similarly, the small overall sample reduces statistical power and may have prevented the detection of subtle but potentially meaningful effects, particularly in subgroup analyses (e.g., by university program, regional area, or BMI category). Additionally, the limited number of participants precluded stratified analyses by individual university programs. This lack of stratification may have masked program-specific variations in stigma levels, further limiting the interpretability of some findings. Another important limitation is the use of self-report measures, which may be subject to social desirability bias, particularly in the assessment of stigma-related attitudes. These limitations underscore the need for larger, more representative studies to further elucidate these relationships and confirm our preliminary findings. Future research should also incorporate strategies to reduce self-reporting bias and include more objective assessments where possible. Importantly, our findings support the implementation of educational interventions aimed at reducing weight stigma in healthcare training programs. Integrating obesity-related stigma awareness, health literacy, and empathic communication into medical and nutrition curricula may help foster more inclusive and effective future healthcare practices.

## Conclusions

In conclusion, our study is the first in Europe to explore attitudes toward individuals with obesity among future healthcare professionals (including medical, healthcare, and nutrition students) while evaluating potential sex based differences in stigmatising attitudes and the influence of health literacy acquired through education. Our findings suggest that weight stigma is not confined to the domain of nutrition therapy but may extend across multiple professional and social contexts, including the broader healthcare sector and sports environments. This highlights the need to assess and address weight stigma comprehensively, beyond nutrition alone, in order to better understand and mitigate its widespread effect.

The presence of weight stigma across educational and clinical settings points to the importance of integrating targeted training programs not only for future nutrition professionals but also for a broader range of healthcare providers and educators. Expanding knowledge about the complex etiopathology of obesity across academic curricula may help reduce stigma, foster empathy, and promote more effective, non-judgmental care. Focusing solely on nutrition therapy risks overlooking other critical settings, where stigma can create barriers to care and the adoption of a healthy lifestyle.

Our findings also suggest that sports and physical activity settings may be potential sources of stigma, potentially discouraging individuals with obesity from engaging in beneficial exercise. Addressing weight stigma in these areas is essential to create inclusive, supportive environments that encourage participation in physical activity, a cornerstone of obesity management and prevention. Therefore, educational interventions should also involve future sports professionals and physical activity instructors, equipping them with the understanding and tools to reduce stigma and to provide holistic support for individuals with obesity. Based on these findings, specific educational strategies should be implemented within healthcare curricula, such as: (1) integrating modules on the multifactorial causes of obesity (including genetic, environmental, and psychological components); (2) offering training in empathic communication and bias awareness; and (3) including testimonials or patient-led sessions that challenge stereotypes and promote humanised perspectives on individuals living with obesity. These approaches have shown promise in previous interventions and may contribute to reducing weight stigma among healthcare trainees. Finally, this pilot study highlights the need for broader research encompassing diverse populations and settings to better delineate the complex relationships between weight stigma, education, physical activity, and healthcare engagement. Future research should include larger samples as well as qualitative studies to explore the underlying beliefs and attitudes that sustain stigma. By expanding the scope beyond nutrition therapy, future studies and interventions can more effectively address the multifaceted impact of weight stigma, ultimately improving access to care, promoting mental and physical health, and fostering equitable treatment for individuals living with obesity.

## Data Availability

The datasets used and/or analysed during the current study are available from the corresponding author on reasonable request.

## Abbreviations

BMI: Body mass index
I-ATOP: Italian version of attitudes toward obese persons questionnaire
SD: Standard deviation
OR: Proportional odds ratio
Cis: Confidence intervals
